# An Overview of CRISPR-Based Tools and Their Improvements: New Opportunities in Understanding Plant–Pathogen Interactions for Better Crop Protection

**DOI:** 10.3389/fpls.2016.00765

**Published:** 2016-06-01

**Authors:** Abdellah Barakate, Jennifer Stephens

**Affiliations:** Cell and Molecular Sciences, The James Hutton InstituteDundee, UK

**Keywords:** CRISPR/Cas9, gene editing, plant–pathogen interactions, DNA double-stranded break, homologous recombination, non-homologous end joining

## Abstract

Modern omics platforms have made the determination of susceptible/resistance genes feasible in any species generating huge numbers of potential targets for crop protection. However, the efforts to validate these targets have been hampered by the lack of a fast, precise, and efficient gene targeting system in plants. Now, the repurposing of clustered regularly interspaced short palindromic repeats (CRISPR)/CRISPR-associated protein 9 (Cas9) system has solved this problem. CRISPR/Cas9 is the latest synthetic endonuclease that has revolutionized basic research by allowing facile genome editing in prokaryotes and eukaryotes. Gene knockout is now feasible at an unprecedented efficiency with the possibility of multiplexing several targets and even genome-wide mutagenesis screening. In a short time, this powerful tool has been engineered for an array of applications beyond gene editing. Here, we briefly describe the CRISPR/Cas9 system, its recent improvements and applications in gene manipulation and single DNA/RNA molecule analysis. We summarize a few recent tests targeting plant pathogens and discuss further potential applications in pest control and plant–pathogen interactions that will inform plant breeding for crop protection.

## Introduction

Genetic crosses and mutagenesis based breeding are time consuming and laborious. The recent development of next generation sequencing is making available fast and cost effective genomic platforms of an increasing number of species including pests, plant models and crops. Now it is easier than ever to perform genome-wide association studies and determine the genes and pathways involved in any particular aspect of pathogen resistance (Olukolu et al., 2014), and pipelines are now well established for genomics-informed breeding (Varshney et al., 2015). It is also quicker and cheaper to obtain the transcriptome of any crop under pathogen attack and determine the virulence and defense pathways and genes that are deployed by both (Kawahara et al., 2012; O’Connell et al., 2012). Proteomics is also starting to make a dent in understanding plant–pathogen interactions (Lodha et al., 2013). A complex network of nuclear processes regulating gene expression and function is emerging from this gene discovery phase but association of a particular pathogen effector with the corresponding host target(s) is still poorly understood (Motion et al., 2015).

Omics technologies generate a huge amount of data and require powerful computational tools to integrate these high-throughput platforms in order to fully understand the multi-layered networks of biomolecules underpinning plant–pathogen interactions. Gene silencing has been extensively used to validate the function of candidate host resistance genes (Duan et al., 2012) and pathogen virulence factors (Yin et al., 2015). However, pathogens have evolved effective suppressors against host RNA silencing, making the system unsuitable for engineering strong and durable resistance in crops (Pumplin and Voinnet, 2013). A more attractive option is gene targeting (GT) which allows any endogenous gene to be disrupted or replaced with a copy that has been manipulated *in vitro*. In GT experiments, double-stranded break (DSB) at the target gene is repaired by one of the two main competing DNA repair pathways: the more frequent non-homologous end joining (NHEJ) pathway or the rare but precise homologous recombination (HR) (Chapman et al., 2012). GT could have a huge impact as a ‘clean transgenesis’ technology for precise gene manipulation or transfer of novel traits into crops. Despite huge efforts, this powerful tool has been elusive in plant science for a long time as it relied on extremely rare spontaneous DSBs (Puchta and Fauser, 2013). However, this barrier has been recently overcome by the development of novel endonucleases that break DNA specifically at chosen genomic targets. Unfortunately, gene replacement by HR is still inefficient in plants and will need further improvement.

Initially, two endonucleases were engineered by fusing a programmable DNA-binding domain to the cleavage domain of the bacterial restriction enzyme *Fok*I. The first endonuclease was generated by linking the DNA-binding domain of a zinc-finger transcription factor to make the first truly flexible chimeric nuclease zinc-finger nuclease (ZFN) (Smith et al., 2000). Similarly, the DNA-binding domain of a transcription activator-like effector of the plant pathogen *Xanthomonas* was used to make the second, and relatively easier to design, nuclease transcription activator-like effector nuclease (TALEN) (Christian et al., 2010). These two big breakthroughs were superseded by an even simpler system based on the clustered, regularly interspaced, short palindromic repeats (CRISPR) and CRISPR-associated genes (Cas) used by some bacteria and Archaea to destroy invading genetic material (Jinek et al., 2012). Our knowledge of CRISPR/Cas is rapidly evolving and the findings are extensively reported and reviewed. Here we will briefly describe the natural and engineered CRISPR–Cas systems followed by the latest and future applications in plant–pathogen interactions.

## The Native CRISPR–Cas System

The CRISPR–Cas system was discovered in bacterial genomes as early as 1987 (Ishino et al., 1987) but its biological role was determined only in 2007 (Barrangou et al., 2007). These evolving adaptive immune systems against invading phages and plasmids are now re-classified into five types I–V (Makarova et al., 2015). During the first invasion, the hosts capture short DNA sequences of about 20 nucleotides, known as spacers, from the foreign genetic material and integrate them between two repeats at the CRISPR locus (Nuñez et al., 2015). Upon subsequent encounters, CRISPR arrays with the acquired spacers are transcribed and processed into small CRISPR RNAs containing the spacer (crRNAs). This chimeric molecule interacts with another auxiliary *trans*-activating CRISPR RNA (tracrRNA), forming a duplex RNA or guide RNA (gRNA) that guides the Cas nuclease to the homologous target (protospacer), resulting in an R-loop structure. The tracrRNA activates Cas nuclease activity, cleaving both strands of the target DNA upstream of a conserved protospacer-adjacent motif (PAM). Cas nuclease has two domains, RuvC and HNH, that cut the PAM-containing strand and its complementary strand, respectively, thus producing a single DSB (Heler et al., 2015). The spacer and PAM requirements depend on CRISPR–Cas type (Xue et al., 2015). In the case of the widely used type II CRISPR–Cas9 system, the last 12 ribonucleotides at the 3′-end of the RNA spacer, known as the seed sequence, dictates the specificity of the complementary target. Mismatches at its 5′-end were thought to be tolerated during gRNA–Cas9 binding to the target. However, the interaction of this region and PAM-distal sequences turned out to be necessary for the activation of Cas9 endonuclease activity (Cencic et al., 2014). PAM sequences are 2–5 bp motifs essential for spacer acquisition and target cleavage (Shah et al., 2013).

## Repurposing of the CRISPR–Cas9 System for Gene Editing in Eukaryotes

The knowledge of the biological function and mechanism of CRISPR–Cas inspired its reprogramming to target any chosen DNA sequence. CRISPR–Cas9 of *Streptococcus pyogenes* was engineered by simply replacing the first 20 nucleotides of crRNA with the intended target sequence and fusing both crRNA and tracrRNA molecules to make a single guide RNA (sgRNA) (Jinek et al., 2012). This newly programmable system was first adopted to target eukaryotic genes in animals, followed by several successful applications in plants including crops (Bortesi and Fischer, 2015; Butler et al., 2015; Lawrenson et al., 2015). The ease of implementation of CRISPR–Cas9 by anyone with basic molecular biology skills has made it the tool of choice for gene editing in any species of interest. Upon generating a DSB at the desired site by the Cas9–gRNA complex, the host cell repairs the DNA lesion by NHEJ pathway resulting in short insertions or deletions, leading to gene knockout. The flexibility of the CRISPR–Cas9 system allows targeting of adjacent sites in a single gene for specific removal of a region, which will be extremely useful for the studies of gene and mRNA *cis*-elements and protein domains (Brooks et al., 2014). CRISPR–Cas9 can also be used in plants to knockout all or single multigene family members (Endo et al., 2015) and even multiple unrelated genes (Lowder et al., 2015).

The DSB lesion can also be repaired by the HR mechanism in the presence of a donor template, leading to precise gene replacement (knock-in). HR-based gene replacement is still inefficient and has been demonstrated in only a few plant species (Bortesi and Fischer, 2015). The efficiency of homology directed repair (HDR) of CRISPR–Cas9 induced DSBs was recently increased by inhibiting the NHEJ pathway in mammalian cells (Chu et al., 2015; Maruyama et al., 2015). Cas9 was recently found to dissociate slowly from DSB by releasing first the 3′end of the cleaved DNA strand that is not complementary to the sgRNA. Consequently HDR was increased to 60% in human cells by using rationally designed single-stranded DNA donor template of the optimal length complementary to the strand that is released first (Richardson et al., 2016). Maize was the first crop where CRISPR–Cas9 was successfully used to generate plants with precise modifications (Svitashev et al., 2015). Precise gene modifications have been achieved at high frequency in tomato by combining the CRISPR–Cas9 nuclease with a geminivirus-based vector for donor DNA template delivery (Čermák et al., 2015). The combination of some or all of the incremental improvements in different animal and plant species could enhance gene replacement efficiency for all crops.

In pathogens, GT without DSB induction was only improved by inhibiting the NHEJ pathway, as in the ku70 mutant of *Verticillium dahliae* (Qi et al., 2015). CRISPR–Cas9 has now made gene editing possible in fungi (Matsu-ura et al., 2015; Nødvig et al., 2015). The effector Avr4/6 of the soybean pathogen *Phytophthora sojae* was efficiently knocked out or even precisely replaced by the selectable marker *npt*II, uncovering additional roles for the corresponding R gene loci *RPS4* and *RPS6* (Fang and Tyler, 2016). The establishment of gene editing tools in *P. sojae* will speed up studies for crop protection in other oomycetes.

Resistance to geminiviruses has been long sought after and was achieved recently in three independent studies using CRISPR–Cas9 in *Nicotiana benthamiana* (Ali et al., 2015; Baltes et al., 2015; Ji et al., 2015). In these works, CRISPR–Cas9 was shown to mutate the viral genome, resulting in reduced viral replication and attenuated infection symptoms. A single gRNA targeting a conserved sequence in the replication origin resulted in efficient inhibition of multiple monopartite and bipartite geminiviruses in the same host. However, further studies will be required to monitor the evolution of this resistance over generations and in more challenging environments (Chaparro-Garcia et al., 2015).

Viral vectors can also be targeted by CRISPR–Cas9 technology to abolish pathogen transmission or even reduce insect population by the so-called mutagenic chain reaction (Gantz and Bier, 2015). This system is initiated when both Cas9 and gRNA transgenes are inserted by homology directed repair at the intended target in males. The transgenes are then copied into the homologous chromosome by HR in the germ-line cells. During fertilization, the males transfer the CRISPR–Cas9 cassette into the next generation and the chain continues. This gene drive system has been demonstrated to be very efficient in manipulating two species of mosquito which are vectors for malaria (Esvelt et al., 2014; Gantz et al., 2015; Hammond et al., 2016). Though attractive, gene drive will not work in self-fertilizing weeds and non-native invasive plant species but it could potentially be used against flies that are vectors of plant pathogens provided that they are amenable to transgenesis. However, safeguarding against the unintended ecological impact of manipulated insect populations is of great importance and biosafety concerns are starting to be addressed by developing antidote systems to reverse gene drive effects (DiCarlo et al., 2015).

## Improvements to the CRISPR–Cas9 System Efficiency and Specificity

Since the conception of the CRISPR–Cas9 gene editing system, its components Cas9 and gRNA have been continuously optimized to improve the efficiency and accuracy of GT (Bolukbasi et al., 2015; Graham and Root, 2015). The repurposing of the CRISPR–Cas9 system to alter eukaryotic genes necessitated targeting the bacterial Cas9 to the nucleus by adding a nuclear localization signal at one or both termini of the protein. To improve translation efficiency, the Cas9 gene was initially codon optimized for human cells and was quickly followed by several plant versions, for both dicots and monocots (Bortesi and Fischer, 2015). The endonuclease Cas9 can easily be converted into a DNA nickase by a single amino acid change in either of its two domains (D10A in RuvC and H840A in HNH; Cong et al., 2013) to cut only one strand. A DSB can still be introduced at the target by these nickases in the presence of two gRNAs that target opposing strands at neighboring sites. This feature has been exploited to improve the specificity of CRISPR–Cas9 and reducing potential off-targets (Ran et al., 2013), a major concern with engineered endonucleases in animals (Hendel et al., 2015) and in plants (Bortesi and Fischer, 2015). Several assays for quantifying on- and off-targets have been developed and inspired strategies for minimizing off-target effects (Hendel et al., 2015; Zhang et al., 2015). In plants, the use of whole genome sequencing as the most accurate method is limited to *Arabidopsis* and rice with good genome reference (Bortesi and Fischer, 2015). Unlike in human gene therapy, off-targets are less problematic in plants where one could eliminate such events by backcrosses. The determination of Cas9 structure (Nishimasu et al., 2014, 2015) has also inspired rational engineering of new Cas9 variants with altered PAM recognition (Kleinstiver et al., 2015) and greater specificity (Slaymaker et al., 2016). Orthologs of commonly used Cas9 from *S. pyogenes* (SpCas9) have been reported to have different features and requirements. The *S. aureus* Cas9 (SaCas9) gene is 1 kbp shorter than SpCas9, improving its stability in viral vectors (Ran et al., 2015). In the screening effort for SpCas9 orthologs, another protein, Cpf1 (CRISPR from *Prevotella* and *Francisella* 1) of type V CRISPR–Cas systems, has been reported to function in a completely different way to Cas9. Cpf1 does not need a tracrRNA but requires a T-rich PAM motif upstream of the target site and generates a DSB with 5′ overhangs (Zetsche et al., 2015).

The design of guide RNAs for efficient and specific gene editing has also been the focus of many studies combining experimental and computing analyses. Several user-friendly algorithms have been developed and freely shared online with the scientific community^1^. Most of these bioinformatics tools are designed to score the efficiency of all potential targets with a PAM motif in the input gene sequence (Wiles et al., 2015). The chance of off-target effects elsewhere in the genome can also be accounted for where the genome sequence is available. These bioinformatics tools are continuously being refined with the availability of new experimental data (Malina et al., 2015; Wong et al., 2015). The structure of the artificial single guide RNA has been revisited recently and improved by lengthening the duplex crRNA/tracrRNA and improving gRNA transcription by shortening its thymine repeat (Dang et al., 2015).

Several systems for delivering Cas9 and gRNA molecules into the cell are available, depending on the species of interest. Plasmid constructs are often used to express Cas9 from RNA polymerase II-driven promoters and gRNAs with polymerase III-mediated transcription. A new strategy based on endogenous tRNA maturation has been developed for expressing multiple gRNAs from a single pol III promoter (Lowder et al., 2015; Xie et al., 2015). While a pol II promoter can be chosen to drive tissue-specific expression of Cas9, snoRNA U3, and U6 pol III promoters are constitutive. However, newly reconstructed sgRNAs can now be expressed from pol II promoters (Wang et al., 2015). Inducible promoters can also be used to induce gene editing *in vivo*, yet reduce potential off-target effects and Cas9-associated toxicity (Dow et al., 2015). Even better, Cas9 and the gRNA can be simultaneously expressed from a single promoter allowing for more spatio-temporal control of each component (Yoshioka et al., 2015). These conditional gene editing methods present new opportunities in crop research but have not yet been tested in plants.

## Other Facets of the CRISPR–Cas9 System

The CRISPR–Cas9 system has become the tool of choice for gene manipulation owing to its simplicity and the willingness of researchers to share the necessary plasmids and methods, including the various algorithms for designing gRNAs. Most of these ingredients are now deposited with the non-profit plasmid repository Addgene^2^ (Harrison et al., 2014). Although most studies focus on knocking out a single gene or a combination of a few targets (multiplexing), the CRISPR–Cas9 system is so powerful that it has been successfully used for genome-wide mutagenesis in mammalian (Malina et al., 2014; Peng et al., 2015) and *Drosophila* (Bassett et al., 2015) cells. The CRISPR–Cas9 based genetic screen uses thousands of unique gRNAs covering the genome of interest and relies on efficient delivery of Cas9/gRNA cargo. This type of forward genetic screen will be very useful in studies of plant–pathogen interactions, but the transformation of plant or pathogen cells must be optimized. This goal can be achieved with at least some pathogens and plant models like *Arabidopsis* and tobacco.

When both RuvC and HNH nuclease domains are mutated, Cas9 becomes an inactive or dead endonuclease (dCas9). Qi et al. (2013) were the first to demonstrate that dCas9 can specifically repress gene expression in *Escherichia coli* in the presence of the gene specific gRNA. This work was quickly followed by another report where dCas9 was fused to transcriptional effectors to silence or activate gene expression in eukaryotes, thereby reversibly manipulating gene expression (Gilbert et al., 2013). Similarly, the epigenome can be manipulated at a specific site by fusing dCas9 with various DNA effectors or histone methylases and acetylases (Hilton et al., 2015; Laufer and Singh, 2015). dCas9-based gene regulation platforms can be used for both genome-wide loss-of-function and gain-of-function screens and the system is amenable to controlled induction (Dominguez et al., 2016). When tagged with fluorescent proteins, dCas9 can be used instead of fluorescence *in situ* hybridization (FISH) to detect chromosomal loci in living (Chen et al., 2013) and fixed (Deng et al., 2015) cells. In this application, dCas9-fluorescent protein fusions can be targeted by a gRNA to a specific locus in the genome for cytological detection. The simultaneous detection of multiple loci in the same cell is feasible by simply fusing different dCas9 orthologs with different fluorescent proteins. Most of the dCas9-based tools will be very useful in deciphering plant–pathogen interactions. Inducible activation or inhibition of master regulators could have huge practical agronomical applications but the down-side is that the dCas9/gRNA transgenes must be kept permanently in the plant.

## Concluding Remarks and Outlook

Different omics platforms have opened the flood gate of potential disease resistance genes that need a more efficient validation pipeline than earlier gene manipulation tools like gene silencing. Plant–pathogen omics data could be improved even further by reducing the background noise in the biological samples. This can now be achieved, for example, by performing cell-type specific RNA or chromatin profiling with novel tools like INTACT (Deal and Henikoff, 2010). Cell-type enrichment will help monitor the dynamics of post-translational modifications during plant–pathogen interactions (Park and Yun, 2013; Motion et al., 2015). CRISPR–Cas9 technology has revolutionized gene manipulation capabilities in many species including crops. The multitude of functions that can be performed with CRISPR–Cas9 and its many derivatives (Sander and Joung, 2014) make it a molecular tool that will open new opportunities in the complicated world of plant–pathogen interactions and help design durable crop resistance to pathogens. Only the gene editing function of CRISPR–Cas9 has so far been used in plants and pathogens. However, the future use of dCas9-based tools will also help to unmask the master regulators of disease resistance (Seo and Choi, 2015). GT tools will help integrate omics data in order to fully understand and improve crop defense mechanisms. The complexity of the plant microbiome with good and bad microbes is beginning to be unraveled (Bai et al., 2015). CRISPR–Cas9 tools will help future studies of plant–pathogen interactions to transcend individual genes or pathogens and become more holistic in approaches to elucidate plant microbiome systems.

## Author Contributions

All authors listed, have made substantial, direct and intellectual contribution to the work, and approved it for publication.

## Conflict of Interest Statement

The authors declare that the research was conducted in the absence of any commercial or financial relationships that could be construed as a potential conflict of interest.

## Footnotes

1 http://omictools.com/crispr-cas9-category

2 https://www.addgene.org/crispr/
